# Modulation of the N170 with Classical Conditioning: The Use of Emotional Imagery and Acoustic Startle in Healthy and Depressed Participants

**DOI:** 10.3389/fnhum.2016.00337

**Published:** 2016-06-30

**Authors:** David A. Camfield, Jessica Mills, Emma J. Kornfeld, Rodney J. Croft

**Affiliations:** ^1^School of Psychology, University of Wollongong, WollongongNSW, Australia; ^2^Illawarra Health and Medical Research Institute, WollongongNSW, Australia

**Keywords:** classical conditioning, event-related potentials, N170, facial processing, major depressive disorder, acoustic startle, affective neuroscience, evaluative conditioning

## Abstract

Recent studies have suggested that classical conditioning may be capable of modulating early sensory processing in the human brain, and that there may be differences in the magnitude of the conditioned changes for individuals with major depressive disorder. The effect of conditioning on the N170 event-related potential was investigated using neutral faces as conditioned stimuli (CS+) and emotional imagery and acoustic startle as unconditioned stimuli (UCS). In the first experiment, electroencephalogram was recorded from 24 undergraduate students (*M* = 21.07 years, *SD* = 3.38 years) under the following conditions: (i) CS+/aversive imagery, (ii) CS+/aversive imagery and acoustic startle, (iii) CS+/acoustic startle, and (iv) CS+/pleasant imagery. The amplitude of the N170 was enhanced following conditioning with aversive imagery as well as acoustic startle. In the second experiment, 26 healthy control participants were tested (17 females and 9 males, age *M* = 25.97 years, *SD* = 9.42) together with 18 depressed participants (13 females and 5 males, age *M* = 23.26 years, *SD* = 4.01) and three conditions were used: CS+/aversive imagery, CS+/pleasant imagery, and CS-. N170 amplitude at P7 was increased for the CS+/aversive condition in comparison to CS- in the conditioning blocks versus baseline. No differences between depressed and healthy participants were found. Across both experiments, evaluative conditioning was absent. It was concluded that aversive UCS are capable of modulating early sensory processing of faces, although further research is also warranted in regards to positive UCS.

## Introduction

Classical conditioning has a long tradition of use in experimental psychology. This paradigm, in its most basic form, involves the learning of an association between a previously neutral stimulus (the conditioned stimulus, CS+) and an unconditioned stimulus (UCS) that is inherently appetitive or aversive. From an evolutionary perspective, it is vital for an individual to rapidly process physical stimulus properties associated with threat (Pizzagalli et al., 2003). For this reason, it is not surprising that fear conditioning can modulate early stimulus-related neural activity within secondary or associative sensory regions within the first 300 ms post-stimulus (Miskovic and Keil, 2012). These changes in sensory processing are attributable to re-entrant modulation of sensory regions by a network of affective brain areas including the amygdala, insula, anterior cingulate, orbital, and lateral pre-frontal cortex (Sehlmeyer et al., 2009; Pessoa and Adolphs, 2010).

Faces are stimuli that are processed in a unique way by the human brain, due to the adaptive importance of rapidly recognizing other humans, and potentially avoiding them in the case of threat. The N170 is an event-related potential (ERP) component that has been found to be strongly associated with facial processing, forming a negative potential at 140–200 ms post-stimulus that is greatest over occipito-temporal sites in the right hemisphere (Posamentier and Abdi, 2003; Graewe et al., 2012). The N170 in response to faces has been found to originate from bilateral sources in the posterior superior temporal sulcus, and is thought to be related to supplementary activity specific to facial processing (Itier and Taylor, 2004). The N170 may also be elicited in response to other face-like stimuli such as greebles (e.g., Levita et al., 2015), or words and other objects, although a larger amplitude is typically observed specifically in response to faces (Mercure et al., 2011).

Previous research suggests that a number of ERP components across a wide latency range may be modulated by emotional stimuli. For example, the late positive potential (LPP) is reliably enhanced in response to emotionally arousing imagery (Olofsson et al., 2008). Emotional faces have also been found to modulate ERP components including P1, N250, early posterior negativity, P300 and the LPP (Vuilleumier and Pourtois, 2007). Whilst the N170 was originally interpreted as a marker of structural encoding related to faces (Labuschagne et al., 2010; Lee et al., 2010; Johnston et al., 2015; Levita et al., 2015), more recent evidence suggests that the N170 may also be sensitive to the emotion displayed in facial expressions (Batty and Taylor, 2003; Blau et al., 2007; Babiloni et al., 2010; Lee et al., 2010; Rellecke et al., 2013). There is some evidence to suggest that the N170 is enhanced specifically for fearful faces (Campanella et al., 2002; Batty and Taylor, 2003; Blau et al., 2007), although other studies suggest that N170 modulation may not be related to specific emotional expressions (e.g., Ashley et al., 2004).

In regards to changes to the N170 in clinical groups it is noteworthy that studies in patients with schizophrenia as well as Huntington’s disease have reported decreased N170 amplitudes, although the decreases were not modulated by facial emotion (Campanella et al., 2006; Croft et al., 2014). However, in regards to depression, Feuerriegel et al. (2015) found N170 amplitude to be unchanged in three out of four studies reviewed, although amplitude was reduced in one study (Dai and Feng, 2012). Similar non-significant findings have also been reported in studies which have investigated manipulations to the serotonergic system, the neurotransmitter most often implicated in depression pathophysiology. Jaworska et al. (2010) found N170 amplitude to be unchanged with acute tryptophan depletion, using neutral, sad, joyful, and surprised faces, and Labuschagne et al. (2010) failed to find alterations with acute serotonin enhancement selective serotonin reuptake inhibitor (SSRI), which would be expected to affect emotional processing. However, the latter study also found that the N170 was enhanced by acute noradrenalin administration serotonin noradrenaline reuptake inhibitor (SNRI), which provides some support for the N170 being modulated by changes in emotional state.

Whilst these previous studies have addressed changes to N170 amplitude as a result of emotional facial expression or emotional state, an unresolved question is whether N170 amplitude may also be altered as a result of conditioning. Electrophysiological measures have been included in a number of recent human fear conditioning studies. These have used a range of aversive UCS including electric shock (Skrandies and Jedynak, 2000), aversive noises (Pizzagalli et al., 2003; Dolan et al., 2006), unpleasant odors (Steinberg et al., 2012), and disturbing imagery (Stolarova et al., 2006). Some of the studies have utilized faces as CS+, either fearful (Pizzagalli et al., 2003), angry (Dolan et al., 2006), or neutral (Steinberg et al., 2012). However, a variety of other CS+ have also been investigated, including Landolt rings (Skrandies and Jedynak, 2000), Gabor gratings (Stolarova et al., 2006; Keil et al., 2007) and “greebles” (Levita et al., 2015). Modulation of ERP components in the same latency range as the N170 has been reported for the studies which utilized facial stimuli. For example, Dolan et al. (2006) found a peak in responses to conditioned angry faces at around 150 ms post-stimulus. Similarly, Steinberg et al. (2012) reported increased magnetoencephalography (MEG) neural activity in occipito-parieto-temporal regions from 130 to 190 ms for faces paired with aversive odors, and in a recent study by Levita et al. (2015), which used instrumental conditioning, the N170 was found to be enhanced in response to face-like greebles that were paired with aversive noise. Also, of note in the study by Levita et al. (2015) was that aversive conditioning resulted in a greater enhancement of the N170 in the right compared to the left hemisphere.

In addition to addressing N170 changes related to conditioning in healthy participants, the question also remains as to whether N170 conditioning is altered as a result of mood disorders, such as major depressive disorder (MDD). According to the neuroplasticity hypothesis of depression (Castrén, 2005; Nissen et al., 2010), experience-dependent modulation of synaptic strength is altered across a number of brain regions. Normann et al. (2007) provided evidence of reduced long-term potentiation (LTP) and altered visual evoked potentials in human visual pathways. However, in regards to the processing of emotional information, there is contrary evidence suggesting LTP enhancement in certain brain regions. Vouimba (2006) reported increased LTP in the amygdala in response to predator stress, whilst a recent study by Nissen et al. (2010) in a sample of severely depressed participants found increased skin conductance response (SCR) to geometric shapes paired with electric shock.

In the current study, changes to N170 amplitude were investigated across two experiments, using differential classical conditioning. The first experiment in healthy participants utilized both visual (aversive or pleasant imagery) and auditory (aversive noise burst) stimuli as UCS, and neutral faces as CS+. In the second experiment in both healthy and depressed participants, aversive and pleasant imagery were used as UCS, and neutral faces were used as CS+. The second experiment addressed two methodological limitations of the first study, by including a baseline measure of N170 amplitude for each CS+ and CS- condition, as well as counter-balancing of faces to different conditions across participants. It was hypothesized that enhancement of N170 amplitude would occur for faces associated with aversive UCS in both experiments, and that the effect would be enhanced for participants with MDD in the second study. In relation to conditioning using pleasant imagery as UCS, no specific predictions were made, as this investigation was exploratory in nature. Subjective ratings of pleasantness for each of the CS+ were also determined before and after conditioning, in order to examine the relationship between electrophysiological change and evaluative conditioning. It was expected that changes to subjective ratings post-conditioning (evaluative conditioning) would be in line with electrophysiological change.

## Experiment 1

### Methods

#### Participants

Thirty-four right-handed participants aged between 18 and 40 years were recruited via the University of Wollongong student research participation website. They received course credit in exchange for participation in the study. However, only 24 of these [12 females, aged 18–33 years (*M* = 21.07 years, *SD* = 3.38 years)] are included in this report, with 10 excluded due to electroencephalogram (EEG) recording issues and/or artifact contamination. All participants were screened according to the following inclusion criteria: No history of traumatic brain injury or neurological illness, normal or corrected-to-normal vision, not currently taking or having taken in the past 30 days pharmaceutical substances or dietary supplements with known effects on cognition and mood, no history of substance abuse or alcoholism and no history of psychiatric disorder. The Beck Depression Inventory-II (BDI-II; Beck et al., 1996) was administered in order to measure the severity of self-reported depressive symptoms. The BDI-II score range was from 0 to 23, indicating that participants were experiencing depressive symptoms that placed them in the minimal to low-moderate range on this instrument. The mean BDI-II score for the group as a whole was 7.67, which was indicative of minimal depressive symptoms within the sample. The study received ethical clearance from the University of Wollongong, Social Sciences Human Research Ethics Committee (approval number HE14/027), and all participants provided written informed consent. All testing was conducted at the Illawarra Health and Medical Research Institute, University of Wollongong from April to November 2014. A list of demographic details is provided in **Table 1.**

**Table 1:** Experiment 1 sample demographics.

	Male (*N* = 12)		Female (*N* = 12)		All participants (*N* = 24)	
	*M*	*SD*	*M*	*SD*	*M*	*SD*
Age	21.04	2.69	21.10	4.08	21.07	3.38
BDI-II	6.42	6.69	8.92	4.25	7.67	5.63
PANAS positive	30.75	3.79	31.25	6.72	31.00	5.34
PANAS negative	19.25	3.02	18.92	3.96	19.08	3.45
NART total errors	13.00	6.41	12.00	4.77	12.50	5.55
FSIQ	117.21	5.32	118.04	3.96	117.63	4.61

*BDI-II, Beck Depression Inventory II; PANAS, Positive and Negative Affect Schedule; NART, National Adult Reading Test; FSIQ, Full Scale Intelligence Quotient (estimated from NART score).*

#### Procedure

On the day of testing participants were additionally required to have abstained from alcohol for 24 h prior and from caffeinated beverages from 10 pm the previous evening. Prior to beginning the experimental task participants completed the subjective face rating task on a DELL laptop, using a mouse to “click and drag” responses on a sliding scale. Participants were seated approximately 80 cm from a DELL 19-inch LCD computer monitor and fitted with a 64 channel stretch lycra Ag/AgCl electrode cap (QuikCap, Compumedics) which contained all of the international 10–20 positions. The electrooculogram (EOG) was recorded from four facial electrodes positioned above and below the left orbit vertical EOG (VEOG) and adjacent to the left and right outer canthus of each eye horizontal EOG (HEOG). EEG was also recorded from the left and right mastoids, for the purposes of oﬄine re-referencing. Neuroscan Acquire Software and a SynAmps2 amplifier (Compumedics, Melbourne, Australia) were used for all EEG recording. Neuroscan Stim2 software was used for all stimulus presentation, with the Stim2 Auditory System P/N 1105 used for delivery of auditory stimuli (Compumedics, Melbourne, Australia). It was ensured that all electrode impedances were below 5 kΩ at the beginning of recording. The participant was grounded by a cap electrode placed between Fz and Fpz and electrodes were referenced to a central in-cap reference located between Cz and CPz. All data was recorded in DC mode using 16-bit A/D conversion at 1000 Hz.

Four experimental task blocks were administered in total, with each block taking no more than 20 min to complete. A short break was provided between each task block in order for the participant to rest. The entire duration of the experiment was around 90 min. At the completion of the EEG recording session, participants again completed the subjective face rating task on the DELL laptop.

#### Materials

##### Beck Depression Inventory II

The BDI-II (Beck et al., 1996) is a 21-item; self-report inventory designed to measure the severity of depressive symptoms over the previous 2 weeks. Score ranges of 0–13 indicate minimal depression symptoms, 14–19 indicate mild depression, 20–28 moderate depression, and 29–63 severe depression.

##### Positive and Negative Affect Schedule

The Positive and Negative Affect Schedule (PANAS; Watson et al., 1988) consists of two 10-item mood scales which measure positive affect and negative affect, respectively. The PANAS has been shown to have high levels of reliability and validity in a large normative study (Crawford and Henry, 2004).

##### National Adult Reading Test

The National Adult Reading Test (NART; Nelson, 1982; Nelson and Willison, 1991) is a 50-item single word reading test which provides a measure of pre-morbid intelligence quotient. In comparison to behavioral tests of intelligence, the NART is much quicker to administer, and has been found to correlate highly with these measures (Crawford et al., 2001; Bright et al., 2002).

##### International Affective Picture System stimuli

A total of 360 images from the International Affective Picture System (IAPS; Lang et al., 2008) were utilized in the experimental conditioning task. IAPS gender-specific normative data was used to selected images according to three conditions. Eighty low valence/high arousal (LVHA) images were selected as unconditioned aversive stimuli (UCS-negative); mean valence = 2.5, mean arousal = 6.0. Forty high valence/high arousal (HVHA) images were selected as unconditioned pleasant stimuli (UCR-positive); mean valence = 7.0, mean arousal = 6.0. A total of 240 neutral valence/low arousal (NEU) images were selected as neutral control images; mean valence = 5.0, mean arousal = 4.0. Separate image lists were selected for males and females, due to differing normative IAPS ratings according to gender. Further details of the means, standard deviations and ranges for each condition, and gender are presented in Supplementary Table S1.

##### NIMSTIM face ratings

Five male faces, with neutral facial expression and closed mouth, were selected from the NIMSTIM faces database (20M_NEC_C, 24_M_NE_C, 28M_NE_C, 34_M_NE_C, and 36M_NE_C). At the beginning of the session, prior to beginning the EEG recording, as well at the completion of the experiment, participants were required to rate each face on a 100-point sliding scale ranging from “extremely unpleasant” (0) to “extremely pleasant” (100). The task was programmed and displayed using PsychoPy (v1.78.00). This rating task was included as a measure of evaluative conditioning.

##### Experimental conditioning paradigm

Five UCS-face pairing conditions were included in the study as follows: (1) CS+/LVHA: LVHA imagery condition, (2) CS+/LVHA + startle: LVHA imagery and acoustic startle condition, (3) CS+/startle only: acoustic startle condition, with neutral IAPS imagery, (4) CS+/HVHA: HVHA imagery condition, and (5) CS-: neutral imagery control condition. Four conditioning blocks were presented across the experiment, with 200 trials presented in each block (800 trials in total). Within each block, 160 CS+ (40 trials in each of LVHA, HVHA, LVHA + startle, startle only) and 40 CS- trials were presented, with a 50% reinforcement schedule applied to all CS+ conditions. This resulted in 320 CS+/paired, 320 CS+/unpaired and 160 CS- trials across the whole experiment (refer to Supplementary Table S2 for further details). For each block separate batches of IAPS images were used, with each image presented twice per CS+ condition and four times per CS- condition. The order of presentation of conditions within each block was pseudo-random, with the constraint that the same condition could not be presented twice consecutively. Two separate sets of randomized blocks (A and B) were created for males and females, and the selection of the block as well as the order of presentation of each of the four blocks was randomized across participants according to a Latin square.

**FIGURE 1:**
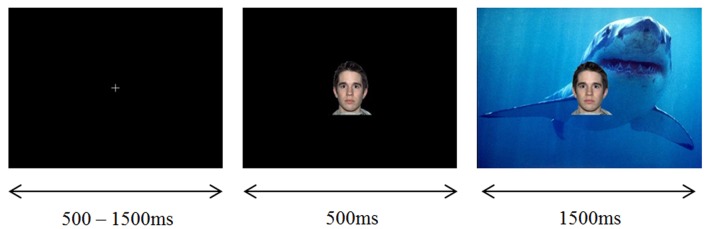
**Example stimuli presentation sequence (LVHA imagery condition)**.

In the experimental task, each trial began with a white fixation cross presented on a black background in the middle of the screen for 500–1500 ms. Following this, a neutral face (CS+ or CS-) was presented on a black background in the center of the screen for 500 ms. Each face was 225 pixels wide and 270 pixels high, according to a computer monitor with 1024 × 768 resolution. 500 ms following the presentation of the face, an IAPS image (UCS) was presented behind the face, replacing the black background (while the same face remained in the foreground). The IAPS image background, together with the neutral face foreground, were presented together for 1500 ms, before being replaced with a white fixation cross on a black background for the start of the next trial. In trials involving the acoustic startle, the startle probe was administered from 1000 to 1200 ms following the presentation of the IAPS image with face overlay, and the offset time of the image was not altered. The acoustic startle consisted of a white noise sound burst delivered binaurally at 103 dB through Sennheiser HD 280 professional high passive noise attenuation headphones. In order to ensure visual vigilance was maintained, the fixation cross was presented in the color red on 10% of trials. When this occurred the participant was required to press the response pad as quickly as possible with their right hand. An example, stimuli presentation sequence is displayed in **Figure 1.**

#### Signal Processing

Oﬄine EEG analysis was performed using Scan 4.5 (Compumedics, Melbourne, Australia). All data were re-referenced oﬄine to linked mastoids and ocular artifact reduction was applied according to the revised aligned-artefact average (RAAA) method (Croft and Barry, 2000). The data was then band-pass filtered from 0.5 to 45 Hz using a zero phase-shift filter (24 dB/octave roll-off). All face stimuli associated with each of the five conditions (both paired and un-paired in the case of CS+ conditions) were epoched from –200 to +500 ms post-stimulus. Epochs were then baseline-corrected according to the pre-stimulus interval, and any epochs found to have amplitudes from any EEG channel that exceeded +/-100 μV were excluded from further analysis. All participants were found to have at least 35 artefact-free epochs (out of a total of 40) in each block contributing to each stimulus condition Averages were then created for each block, condition and participant.

#### N170 Scoring

N170 scoring was restricted to electrodes P7 and P8. In order to better facilitate the detection of peak components relevant to facial processing each of the selected channels was re-referenced to its corresponding (reversed polarity) frontal channel (P7-F7, P8-F8; Croft et al., 2014). The N170 was defined as the most negative point in the range from 130 to 210 ms. Peak picking was conducted for ERP data averaged across the entire experiment (blocks 1–4), as well as separately for the first half of the experiment (blocks 1 and blocks 2 combined; time 1) and the second half of the experiment (blocks 3 and blocks 4 combined; time 2). The latter was conducted in order to investigate change to N170 amplitudes over the course of the conditioning paradigm.

#### Statistical Analysis

For the analysis of evaluative conditioning, a mixed model repeated-measures ANOVA was conducted on change scores for pleasantness ratings after conditioning in comparison to change scores before conditioning. A subject-specific random intercept term was fitted, together with the fixed effect of stimulus condition (control, LVHA, LVHA + startle, startle, HVHA), with pleasantness rating before conditioning included as a covariate. Effect size was calculated using estimated marginal means and presented as Cohen’s *d*. For the analysis of N170 peak amplitude data across all blocks, a repeated-measures ANOVA was conducted on the N170 peak amplitudes, with condition (LVHA, LVHA and startle, startle, HVHA, control) and laterality (P7, P8) as within-subject variables. Preliminary analysis regarding the effect of gender on N170 amplitudes did not indicate any main effects or interactions with other variables, and for this reason it not included in subsequent analysis. As an additional analysis to investigate the change in N170 across the course of the experiment, a repeated-measures ANOVA was conducted on the change in N170 peak amplitude from time 1 to time 2, with condition (LVHA, LVHA and startle, startle, HVHA, control) and laterality (P7, P8) as within-subject variables. Effect size was presented as partial η^2^.

For all behavioral and ERP measures, planned contrasts were used to compare values between the control condition (CS-) and each of the other conditions separately (CS+/LVHA, CS+/LVHA and startle, CS+/startle, and CS+/HVHA). In order to assess the relative efficacy of the three aversive conditioning methods (CS+/LVHA, CS+/LVHA + startle, and CS+/startle only), repeated-measures ANOVAs were conducted using difference scores calculated between the CS- condition and each of CS+/LVHA, CS+/LVHA+startle, and CS+/startle (only) conditions. Planned contrasts were then used to compare differences between (i) CS+/LVHA and CS+/LVHA + startle conditions, (ii) CS+/startle and CS+/LVHA + startle conditions, and (iii) CS+/LVHA and CS+/startle conditions. No Bonferroni-type α adjustment was required, due to all contrasts being planned and not exceeding the degrees of freedom for effect (Tabachnick and Fidell, 1989). Similarly, Greenhouse–Geisser type correction was not necessary because single degree of freedom contrasts are not affected by the violations of sphericity assumptions common in repeated-measures analyses (O’Brien and Kaiser, 1985).

### Results

#### Evaluative Conditioning

**FIGURE 2:**
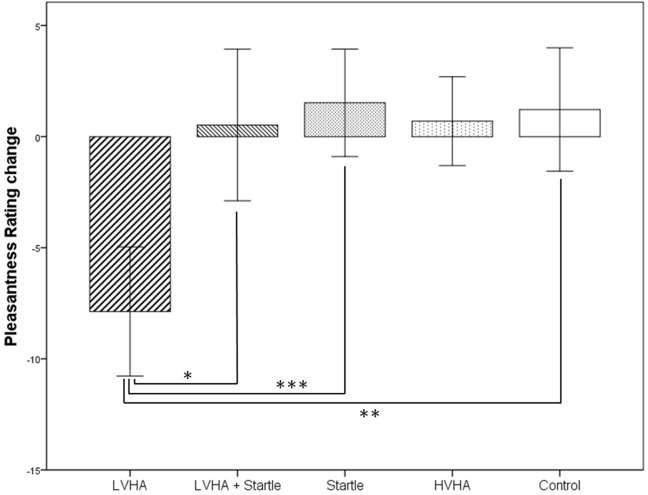
**Change in pleasantness rating from before to after conditioning paradigm, according to stimulus condition (*N* = 23).** ^∗^*p* < 0.05, ^∗∗^*p* < 0.01, ^∗∗∗^*p* < 0.001.

Means and standard deviations of the subjective pleasantness ratings for the faces both before and after the conditioning paradigm are displayed in Supplementary Table S3 according to gender, with change scores displayed in **Figure 2.** Rating data from one participant was unavailable (*n* = 23).

A decreased pleasantness rating was found for the CS+/LVHA condition in comparison to the CS- condition (mean difference = –14.245, *p* = 0.002, *d* = –1.12). Pleasantness ratings were decreased for the CS+/LVHA condition in comparison to the both the CS+/LVHA + startle condition (mean difference = –11.504, *p* = 0.015, *d* = –0.68) and the CS+/startle (only) condition (mean difference = –16.586, *p* < 0.001, *d* = –1.16) post-conditioning.

#### N170 Amplitudes

**FIGURE 3:**
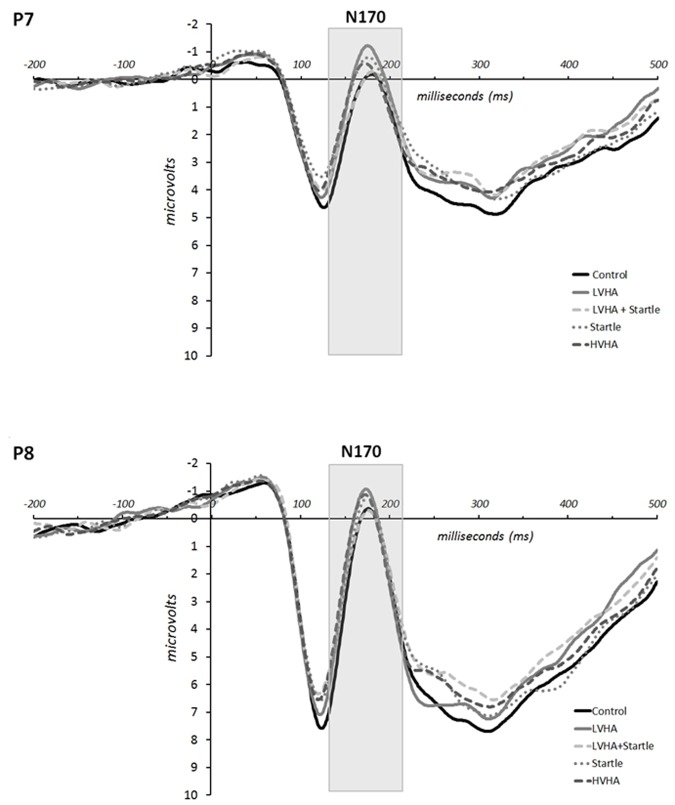
**Experiment 1, grand mean event-related potentials for P7 and P8**.

The grand means for the ERPs according to condition are displayed in **Figure 3** for electrodes P7 and P8. Means and standard deviations of the N170 peak amplitudes are displayed in Supplementary Table S4 according to gender, condition, and time.

N170 amplitude was increased for the CS+/LVHA condition in comparison to CS- [*F*(1,22) = 14.285, *p* = 0.001, partial η^2^ = 0.383], as well as for the CS+/startle (only) condition in comparison to CS- [*F*(1,22) = 13.838, *p* = 0.001, partial η^2^ = 0.376]. The increase in N170 amplitude was greater for the CS+/LVHA condition in comparison to the CS+/LVHA + startle condition [*F*(1,22) = 8.041, *p* = 0.009, partial η^2^ = 0.259]. No significant differences in N170 amplitudes were found between the CS+/LVHA and the CS+/startle (only) conditions.

**FIGURE 4:**
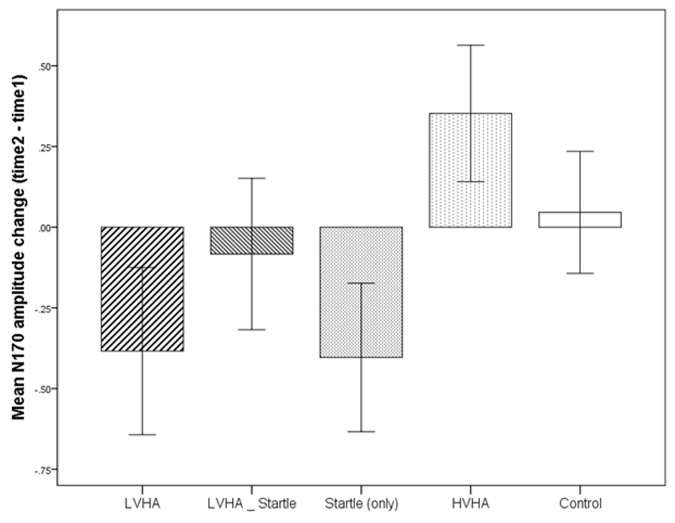
**Experiment 1, change in N170 from time 1 to time 2, according to stimulus condition**.

The changes in N170 amplitude from time 1 to time 2 according to each condition are displayed in **Figure 4.** The condition by time (time 1, time 2) interaction was found to be non-significant. Similarly, differences in N170 amplitudes between the control condition and each of the aversive conditions were not found to be significantly changed from time 1 to time 2. (All main effects and interactions are listed in Supplementary Table S9).

## Experiment 2

### Methods

#### Participants

Forty-four right-handed participants aged between 18 and 50 years were recruited from the University of Wollongong and the greater Illawarra region, New South Wales, Australia. Students who participated in the study received course credits in exchange for their participation, whilst participants from the general public were reimbursed $50 AUD for their time and travel expenses. There were 26 participants in the healthy control group (17 females and 9 males, age *M* = 25.97 years, *SD* = 9.42) and 18 participants in the depressed group (13 females and 5 males, age *M* = 23.26 years, *SD* = 4.01). All participants were screened according to the same general exclusion criteria as Experiment 1, with the exception of depression screening criteria. More detailed screening in regards to alcohol and other recreational substances was also conducted in Experiment 2. Any participant found to be consuming > 20 standard drinks of alcohol per week was excluded, and any participant consuming cannabis or amphetamines more than once per week over the past month was also excluded from participation in the study. For inclusion in the depressed group, participants were required to meet Diagnostic and Statistical Manual of Mental Disorders, Fourth Edition (DSM-IV) criteria for a current depressive episode, as confirmed by the Mini-International Neuropsychiatric Interview (M.I.N.I), version 5.0 (Section A) and to score >19 on the BDI-II, being indicative of at least moderate depression severity. They were not permitted to have any other comorbid psychiatric disorders, with the exception of a personality or anxiety disorder. One participant indicated on the screening questionnaire that they had an existing diagnosis of borderline personality disorder. Stable treatment with antidepressants or psychosocial therapy was also permitted. For inclusion in the healthy control group, absence of a current depressive episode was confirmed using the M.I.N.I. version 5.0 as well as a score on BDI-II < 14, indicative of minimal depression severity. They were also not permitted to have any other psychiatric disorder. The study received ethical clearance from the University of Wollongong, Social Sciences Human Research Ethics Committee (approval number HE15/094), and all participants provided written informed consent. All testing was conducted at the Illawarra Health and Medical Research Institute, University of Wollongong from July to November 2015, and formed part of a larger investigation into electrophysiological correlates of depression. A list of demographic details is provided in **Table 2.**

**Table 2:** Experiment 2 sample demographics.

	Control (*N* = 26)		Depressed (*N* = 18)		All participants (*N* = 44)	
	*M*	*SD*	*M*	*SD*	*M*	*SD*
Age	25.97	9.42	23.26	4.01	24.86	7.73
BDI-II	4.19	3.91	35.44	10.18	16.98	17.07
PANAS positive	29.23	7.71	20.94	6.24	25.84	8.18
PANAS negative	11.92	1.98	22.39	8.60	16.20	7.66
PSS	13.88	4.79	29.78	4.98	20.39	9.25
HAM-A	5.35	4.32	26.56	12.84	14.02	13.69
FSIQ (NART)	112.20	4.00	114.03	4.40	112.95	4.22

*BDI-II, Beck Depression Inventory II; PANAS, Positive and Negative Affect Schedule; PSS, Perceived Stress Scale; HAM-A, Hamilton Anxiety Rating Scale; NART, National Adult Reading Test; FSIQ, Full Scale Intelligence Quotient (estimated from NART score).*

#### Procedure

As per Experiment 1, on the day of testing, participants were asked to abstain from alcohol for 24 h and caffeine from 10 pm the previous evening. Additionally no participant reported using amphetamines, cannabis, or any other psychotropic substances within 5 days of the testing session. Prior to beginning the experimental task participants completed the subjective face rating task on a DELL laptop, using a mouse to “click and drag” responses on a sliding scale. All stimulus display and EEG recording equipment used in the conditioning task were the same as in Experiment 1, with the exception that only 32-channel EEG was recorded in Experiment 2. Three experimental task blocks (baseline, conditioning block 1, conditioning block 2) were administered in total, with each block taking around 10 min to complete. A short break was provided in between each task bloc, with the entire duration of the experiment around 40 min. After completion of the task blocks, participants provided post-conditioning subjective face ratings on the DELL laptop. At the completion of the EEG recording session, a subset of 29 participants, as well as an additional one participant not included in the EEG analysis due to technical issues, provided valence and arousal ratings on a DELL laptop computer for the full set of IAPS images utilized in the conditioning paradigm. There were 30 participants in total who provided ratings (19 controls: 12 females and 7 males; age *M* = 24.84 years, *SD* = 8.70 and 11 depressed: 7 females and 4 males; age *M* = 23.88 years, *SD* = 3.81). Ratings were provided using a computerized version of the self-assessment manikin (Lang et al., 2008).

#### Materials

Beck Depression Inventory II, Positive and Negative Affect Schedule, and National Adult Reading Test as per Experiment 1.

##### The Mini International Neuropsychiatric Interview – Section A

The M.I.N.I for DSM-IV (version 5.0, Sheehan et al., 1998) is a short structured clinical interview designed for use in a research environment, which enables a diagnosis of psychiatric disorders according to DSM-IV criteria. Sections relevant to the diagnosis of MDD, with or without melancholic features (Section A and A’) were used.

##### Hamilton Anxiety Rating Scale (HAM-A)

The HAM-A (Hamilton, 1959) is a 14-item scale designed to assess the severity of cognitive and somatic trait anxiety symptoms. The total score range is from 0 to 56, with <17 indicating mild anxiety, 18–24 moderate anxiety and >25 indicating severe anxiety.

##### Perceived Stress Scale

The Perceived Stress Scale consists of 14 items which have been designed to measure a respondent’s perception of stress. Total scores range from 0 to 56 with higher scores indicating a greater degree of perceived stress and lower scores indicating effective coping.

##### International Affective Picture System stimuli

A total of 120 images from the IAPS (Lang et al., 2008) were selected separately for males and females in the experimental conditioning task for Experiment 2. According to the IAPS gender-specific normative data, 40 LVHA (negative) images were selected as UCS-negative, 40 HVHA (positive) images were selected as UCS-positive, and 40 NEU (neutral) images were selected as neutral images for use in the CS- condition. Mean valence ratings for the neutral, negative, and positive conditions were 5.0, 3.00, and 6.97, whilst mean arousal ratings for neutral, negative, and positive conditions were 3.01, 5.98, and 5.99 (respectively). Means, standard deviations, and ranges for the gender-specific normative valence and arousal values per condition and gender are presented in Supplementary Table S5.

##### NIMSTIM face ratings

Three male faces from the NIMSTIM faces database which had neutral facial expression and closed mouth (20M_NEC_C, 24_M_NE_C, and 28M_NE_C) were retained from Experiment 1. At the beginning of the session, prior to beginning the EEG recording, as well at the completion of the experiment, participants were required to rate each of the three faces, as well as an additional two control faces (34_M_NE_C and 36M_NE_C) on a 100-point sliding scale ranging from “extremely unpleasant” (0) to “extremely pleasant” (100).

##### Experimental conditioning paradigm

Three stimulus-face pairing conditions were included in Experiment 2. The conditions were as follows: (i) CS+/negative: LVHA imagery condition, (ii) CS+/positive: HVHA imagery condition, and (iii) CS-: NEU control condition. A 100% reinforcement schedule was applied for the CS+/negative and CS+/positive conditions, with UCS (LVHA or HVHA images) paired with neutral NIMSTIM faces across all trials. Three blocks were presented in total, consisting of one baseline block with only faces (without associated imagery), and two conditioning block. Eighty images per condition (consisting of 20 images duplicated four times each) were presented in each of the two conditioning blocks, resulting in 480 images across the two experimental task blocks (160 per condition). Mean valence and arousal ratings within each condition were balanced across task blocks. In the baseline block, each NIMSTIM face was presented 80 times each in the absence of associated imagery (240 trials in total). Six different experimental sequences were produced in order to control for the pairing of NIMSTIM face with experimental condition (CS+/negative, CS+/positive, CS-), and the sequences were counterbalanced across participants according to a Latin square.

The timing of stimulus presentation was the same as for Experiment 1, with a white fixation cross appearing on a black background in the middle of the screen for 500–1500 ms, followed by the NIMSTIM face on a black background in the center of the screen for 500 ms, followed by an IAPS picture replacing the black background underneath the NIMSTIM face for 1500 ms. For the baseline block, without IAPS imagery, in each trial the fixation cross appeared for 500–1500 ms followed by the NIMSTIM face for 2000 ms. In order to ensure visual vigilance, the fixation cross was presented in the color red on 10% of trials, with participants required to respond by button press with their right hand.

#### Signal Processing and N170 Scoring

All oﬄine EEG analysis and N170 scoring procedures were the same as in Experiment 1. Peak picking of N170 amplitudes was conducted for each task block separately (baseline, conditioning block 1, conditioning block 2).

#### Statistical Analysis

For the analysis of evaluative conditioning the same mixed model repeated-measures ANOVA was used as per Experiment 1. Image rating was analyzed using separate repeated-measures ANOVAs for valence and arousal ratings. For each test, level (high, low) was the within-subject variable, and gender (male, female) and depression status (control, depressed) were between-subject variables. Planned contrasts were used to compare ratings between the neutral versus negative condition and the neutral versus position condition. Contrasts were also used to compare negative-neutral difference scores versus positive-neutral difference scores. For the analysis of N170 peak amplitude data across all blocks, a repeated-measured ANOVA was conducted on the N170 peak amplitudes, with block (baseline, block 1, block 2), condition (negative, positive, neutral) and laterality (P7, P8) as within-subject variables, and depression status (control, depressed) as the between-subject variable. Effect size was presented as partial η^2^. As per Experiment 1, gender was not included as a variable in the analysis due to preliminary analysis on N170 amplitudes revealing no main effects or interactions with other variables. Planned contrasts were used to compare values between the CS- condition and each of the CS+ conditions (CS+/negative versus CS-, CS+/positive versus CS-), and in order to compare changes in N170 amplitude over the course of the experiment a Helmert contrast was used to compare amplitude at baseline with amplitude in the subsequent conditioning blocks (mean of blocks 1 and 2). In order to assess the relative changes in N170 amplitude as a function of conditioning using CS+/negative versus CS+/positive conditions, repeated-measures ANOVAs were conducted using difference scores calculated between the CS- and the CS+/negative and CS+/positive conditions. Planned contrasts were used to compare differences between negative-neutral and positive-neutral values.

### Results

#### Image Ratings

Means and standard deviations of the mean valence and arousal ratings for each of the IAPS images used in Experiment 2 conditioning paradigm are displayed in Supplementary Table S6 according to condition (negative, positive, neutral), gender (male, female), and group (control, depressed).

Negative images were rated higher in arousal than neutral images [negative > neutral: *F*(1,26) = 47.328, *p* < 0.001, partial η^2^ = 0.645] and positive images were rated higher in arousal than neutral images [positive > neutral: *F*(1,26) = 66.581, *p* < 0.001, partial η^2^ = 0.719]. Overall, arousal ratings were higher for the control group in comparison to the depressed group [control > depressed: *F*(1,26) = 9.141, *p* = 0.006, partial η^2^ = 0.260]. The increased arousal rating for positive in comparison to neutral imagery was larger in the control group [positive > neutral × control > depressed: *F*(1,26) = 5.661, *p* = 0.025, partial η^2^ = 0.179]. No differences between negative-neutral and positive-neutral arousal ratings were found.

Negative images were rated lower in valence than neutral images [negative > neutral: *F*(1,26) = 113.8978, *p* < 0.001, partial η^2^ = 0.814] and positive images were rated higher in valence than neutral images [positive > neutral: *F*(1,26) = 55.981, *p* < 0.001, partial η^2^ = 0.683], with this effect being larger in the control group [positive > neutral × control > depressed: *F*(1,26) = 4.851, *p* = 0.037, partial η^2^ = 0.157]. Positive-neutral valence ratings were higher than negative-neutral valence ratings [positive-neutral > negative-neutral: *F*(1,26) = 154.310, *p* < 0.001, partial η^2^ = 0.856].

#### Evaluative Conditioning

Means and standard deviations of the subjective pleasantness ratings for the faces associated with each condition (CS+/negative, CS+/positive, CS-) both before and after the conditioning paradigm are displayed in Supplementary Table S7 according to gender (male, female) and group (control, depressed). No significant post-conditioning changes in pleasantness ratings were found for faces associated with any of the experimental conditions (CS+/positive, CS+/negative, CS-), and no significant effect for depression status was found (healthy, depressed) either pre- or post-conditioning.

#### N170 Amplitudes

**FIGURE 5:**
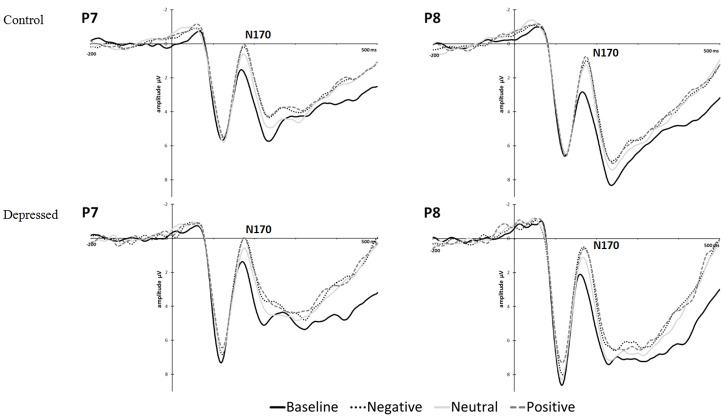
**Experiment 2, grand mean event-related potentials for P7 and P8 by group (control, depressed) and condition for conditioning blocks 1 and 2 combined**.

The grand means for the ERPs according to condition and group (control, depressed) are displayed in **Figure 5** for electrodes P7 and P8. Means and standard deviations of the N170 peak amplitudes are displayed in Supplementary Table S8 according to gender, condition, and time.

No differences in N170 amplitudes were found between depressed and control at baseline (pre-conditioning). Higher amplitude was found for the conditioning blocks in comparison to baseline [conditioning > baseline: *F*(1,42) = 25.355, *p* < 0.001, partial η^2^ = 0.376]. Higher amplitude in the CS+/positive condition was found in comparison to CS- across all blocks [CS+/positive > CS-: *F*(1,42) = 6.456, *p* = 0.015, partial η^2^ = 0.133]. The increase from baseline to conditioning blocks was larger for P8 in comparison to P7 [conditioning > baseline × R > L: *F*(1,42) = 4.669, *p* = 0.036, partial η^2^ = 0.100]. However, a greater increase in P7 amplitude was observed from baseline to conditioning blocks for the CS+/negative condition versus CS- condition [conditioning > baseline × L > R × CS+/negative > CS-: *F*(1,42) = 4.348, *p* = 0.043, partial η^2^ = 0.094]. No significant differences between (CS+/negative—CS-) and (CS+/positive—CS-) conditions were found. (All main effects and interactions are listed in Supplementary Table S10).

## Discussion

### Experiment 1 Conditioning Effects

The primary finding of Experiment 1 was that the pairing of neutral faces with either low valence imagery (LVHA) or acoustic startle resulted in potentiated N170 amplitudes in comparison to the control condition, where faces were unpaired. In relation to enhancement of the N170 for a neutral faces paired with acoustic startle, this finding is consistent with previous research by Levita et al. (2015) whereby N170 amplitude was found to be increased in response to face-like greebles that were associated with a loud aversive noise in an instrumental avoidance task. These findings are also consistent with Dolan et al. (2006) whereby a peak in ventral-occipital activity was observed around 150 ms for Ekman faces paired with aversive noise. Taken together there now appears to be consistent evidence across different studies suggesting enhancement of N170 amplitude for faces paired with aversive noise, with its effectiveness perhaps attributable to it being a particularly strong and unpleasant UCS in comparison to aversive stimuli from other modalities.

In contrast to the significant changes to N170 amplitudes found to occur when acoustic startle was used by itself as an UCS in Experiment 1, no changes to either N170 amplitude or evaluative condition was observed when acoustic startle was combined with aversive imagery. To the best of our knowledge, this is the first time that their combination has been examined in the same study, so clarification of this finding by comparison to previous studies is not possible. However, two possible reasons for the lack of significant findings include (i) a stronger habituation effect over the course of the trial for this condition, and/or (ii) an interference effect whereby the simultaneous presentation of aversive auditory and visual stimuli resulted in insufficient attention being directed to either sensory modality, and hence less effective conditioning. In regards to the possibility of more rapid habituation, the absence of a time by condition interaction when the first half of the experiment was compared to the second half of the experiment, suggests that this is unlikely to account for the finding. However, it cannot be discounted that a “rapid” degree of habituation had already occurred within the first half of the experiment (time 1) for this condition, a possibility that is in need of further empirical investigation.

In relation to the finding of enhanced N170 amplitude in response to the neutral face paired with unpleasant IAPS imagery in Experiment 1, to the authors’ knowledge this is the first time that this has been demonstrated in humans. Whilst there is previous evidence to suggest that the use of IAPS imagery as UCS may result in changes to early sensory (<100 ms) components associated with visual processing (Stolarova et al., 2006; Keil et al., 2007), these studies employed only simple Gabor grating patterns as their conditioned stimuli. For this reason, these previous findings cannot be compared directly with the current ones, which utilized more complex stimuli (neutral faces) as CS+. Nevertheless, the current findings are in line with expectation, and demonstrate that both visual as well as auditory aversive stimuli may result in changes to early facial processing. Interestingly, the CS+/LVHA condition was also the only one found to be associated with significantly decreased subjective pleasantness ratings post-conditioning. The reason for a change in subjective rating for this CS+ and not the CS+ associated with acoustic startle is not immediately apparent. Considering that comparable N170 amplitude changes were observed for both the CS+/LVHA and CS+/startle conditions, it would be expected that equivalent changes in evaluative conditioning would also be observed. A possible explanation is that the combination of UCS and CS+ in the same sensory (visual) modality had a stronger effect on evaluative conditioning than when UCS and CS+ were taken from different sensory modalities (i.e., auditory and visual). However, this account is speculative and is in need of further empirical testing.

In relation to the CS+/pleasant condition, no significant changes were observed for either N170 amplitudes or evaluative conditioning. The finding of no change to the N170 amplitude suggests that the face was not attributed any increase or decrease in salience in comparison to the control condition. Similarly, no change in subjective pleasantness rating was observed for this face. The fact that no increase in subjective pleasantness occurred for this face is in line with the N170 results, however, it is also a possibility that the images were not experienced as being significantly more pleasant than the neutral stimuli in the current sample. Though the IAPS has been validated across a wide range of ages and ethnicities, there is a larger degree of variability in subjective arousal and valence ratings for certain images such as erotica images (Libkuman et al., 2007; Lang et al., 2008). For this reason, it is possible that certain images within this battery may not have been perceived as highly arousing and pleasant within the Australian undergraduate university sample. Unfortunately, valence and arousal ratings for the UCS in the current sample were not available.

There were two significant limitations to the first experiment that were addressed in Experiment 2. The first limitation was in regards to the experimental design, whereby baseline differences in the sensory and emotional processing of the neutral faces may have had a confounding effect on the N170 amplitudes that were observed following conditioning. In particular, the lower baseline pleasantness rating for the neutral face associated with the CS+/LVHA condition may have contributed to the significant N170 change post-conditioning. If increased salience was afforded this face in comparison to the others, then N170 changes may have been occurring independent of those attributable to conditioning. However, a significant decrease in pleasantness ratings post-conditioning, together with a trend (**Figure 4**) toward further increase in N170 amplitude across the second half of the experiment, suggests that this post-conditioning change could not be attributed to baseline differences alone. Nevertheless, this limitation was addressed in Experiment 2, whereby counter-balancing of the faces to different conditions across participants ensured that this confound was controlled for.

The second limitation of Experiment 1 was that no baseline measure of N170 amplitude was provided, and for this reason, it was unknown to what extent amplitudes changed across the course of the experiment, in particular the first half of the experiment. However, what could be determined in the second half of the experiment (time 1 to time 2) was that due to the lack of a significant main effect for time and lack of a significant time by condition interaction, it appeared that N170 amplitudes neither habituated nor increased significantly for the second half of the experiment in comparison to the first half of the experiment. This finding is in contrast to the study by Levita et al. (2015), whereby an overall fatigue/habituation effect was reported across all conditions, with N170 amplitudes being larger in the first compared to the second half of the experiment. Also noteworthy is that, whilst the changes in the aversive conditions from time 1 to time 2 (see **Figure 4**) were non-significant when compared to control, the trend was in line with the main effect for condition, whereby a greater increase in N170 amplitude was observed for both the LVHA and the startle conditions across the second half of the experiment. In Experiment 2, a more detailed analysis of change in N170 responses was made possible by the inclusion of a baseline measure of N170 amplitude for each CS+ and CS- condition.

### Experiment 2 Conditioning Effects

The main finding of Experiment 2 was that N170 amplitude in the left hemisphere increased to a greater degree from pre- to post-conditioning for the negative CS+ condition in comparison to the CS- condition. In regards to the CS+/negative condition, this finding corroborates the finding of increased N170 amplitude that was observed in Experiment 1 for a neutral face paired with aversive imagery. These findings are again in line with reports from previous studies (Stolarova et al., 2006; Keil et al., 2007) whereby modulation of early sensory components was found when aversive IAPS imagery was paired with Gabor grating CS+. In consideration of the fact that Experiment 2 addressed the two primary limitations of the first experiment, namely counterbalancing of faces across conditions and the inclusion of a baseline N170 measure, these findings provide stronger evidence regarding the ability of aversive IAPS imagery to modulate face-specific processing.

Whilst N170 amplitude was not found to be significantly increased in the CS+/positive condition in Experiment 2, it is noteworthy that the difference between CS+/negative and CS+/positive was non-significant (it also can be noted that although non-significant, there was also a trend toward an increase in the CS+/positive condition versus CS- from baseline to conditioning blocks at P7, with *p* = 0.074). For this reason, the possibility remains that conditioning effects were also occurring in association with the positive imagery, albeit the current study may not have had sufficient power to detect it. In consideration of the fact that no previous investigations have been conducted regarding the effect of positive classical conditioning on N170 responses to facial stimuli, further research is warranted.

Whilst an overall right-hemisphere lateralization was found for the N170 across conditions, it is of interest that left hemisphere lateralization was found for N170 response to the CS+/negative condition. This finding is in contrast to the study by Levita et al. (2015), whereby N170 amplitude was enhanced in the right compared to the left hemisphere following aversive operant conditioning. Whilst it is interesting to note that left lateralization of specific facial expression processing has been reported in a number of studies (Borod et al., 2002), these findings have typically been attributable to the specific facial expressions associated with particular emotions. For example, in a recent fMRI study by Calvo and Beltrán (2014), processing of the mouth region in smiling faces was found to be left lateralized for the occipito-temporal region in the latency range of the N170. In contrast, in a visual field study by Anders et al. (2005), conditioning with an aversive scream as the UCS and a neutral face as the CS+ revealed no hemifield advantage for the conditioned face, as evidenced by functional magnetic resonance imaging (fMRI) blood-oxygen-level dependent (BOLD) signal, SCR, and eyeblink startle responses. In light of this previous study, the current finding of enhanced left hemisphere N170 amplitude relative to the right is difficult to reconcile, and further investigation in future studies is warranted.

In regards to interpreting the meaning of N170 enhancement as a result of conditioning, there are two possible accounts, although not necessarily mutually exclusive. The first of these is that as a result of conditioning the faces become warning cues for impending aversive experiences. The putative neural mechanism by which conditioning may alter early facial processing is via re-entrant connections between the amygdala and visual pathways (Vuilleumier, 2005), with previous research suggesting that emotional arousal may play an important role in modulating the degree of amygdala activation during conditioning (McGaugh, 2004). However, other aspects of the ventral emotional pathway such as the insula, ventral striatum, and the anterior cingulate and prefrontal cortex (Phillips et al., 2003a) are also likely to be involved in the modulation of facial processing under conditions of aversive and/or appetitive UCS. An alternative interpretation of the changes to N170 amplitude is that the faces themselves begin to be perceived as unpleasant or pleasant in their own right. However, the lack of evaluative conditioning in the current study argues against this possibility.

### Modulation of Conditioning by Depression Status

The lack of a significant effect for depression status on N170 amplitude is contrary to expectation. Specifically, no support was found for the prediction of increased N170 amplitude for depressed individuals in the CS+/negative condition. Whilst the current study was the first of its kind to investigate the effect of classical conditioning with emotional imagery on facial processing, there is a large body of work which attests to differences in conditioned responses associated with depression using other UCS/CS+ combinations (Vouimba et al., 2006; Normann et al., 2007; Nissen et al., 2010). For instance, using an animal model of chronic stress, Vouimba et al. (2006) reported increased LTP in the amygdala, a finding that is consistent with other reports of increased activity in the ventral emotional system as a consequence of depression (Phillips et al., 2003b).

The fact that no differences in sensory processing of faces were found for depressed individuals in the current study suggests that either re-entrant modulation of sensory regions by the amygdala (Sehlmeyer et al., 2009; Pessoa and Adolphs, 2010) was not sufficiently different following conditioning compared to controls, and/or that amygdala activity was not enhanced in the depressed group. Without neuroimaging data relating to amygdala activation in the current study, it is unclear as to which of these accounts may be accurate. It is also informative to note that no differences in baseline N170 amplitude were observed between depressed and control participants. However, this is consistent with other studies which have investigated ERP responses to faces with neutral expressions in depressed participants (Feuerriegel et al., 2015).

It should also be acknowledged that the lack of significant effect for depression status may also be attributable to symptom heterogeneity within the depressed group. Whilst self-report measures of depression severity such as the BDI-II assume that a single factor explains the majority of variance, this is not an entirely accurate representation of symptom expression, with patients typically presenting with a mixture of cognitive, somatic, and affective symptoms (American Psychiatric Association, 2013). In future studies it would be advantageous to investigate changes in the N170 as a function of specific symptomology, rather than an umbrella grouping based on overall depression severity.

An important issue that has implications for the interpretation of the results is that valence and arousal ratings of the images used as UCS differed between depressed and control groups. Depressed participants provided arousal ratings for the images which were lower than for control, particularly in regards to the positive imagery. Similarly, valence ratings were lower for positive images in the depressed group in comparison to controls. These findings are consistent with the anhedonia observed in depression, characterized by a diminished response to positive material (Sloan et al., 2001). Similar findings were reported by Sloan et al. (2001) whereby depressed women reported feeling less pleasant and less emotionally aroused when viewing positive pictures relative to controls. Dunn et al. (2004) also found a blunted response to positive imagery, with depressed participants reporting both reduced arousal and valence. However, other studies have reported contrary findings, such as Allen et al. (1999) where valence and arousal ratings of IAPS pictures were largely similar for depressed and control individuals, and Pause et al. (2000) where only negative images and odors were rated as higher in arousal by depressed participants. Considering that the positive imagery was experienced as less arousing and pleasant for the depressed individuals in the current study, it could be argued that conditioning for pleasant stimuli was less effective in these individuals. However, the lack of a group interaction for N170 amplitudes suggests that this difference was not of great enough magnitude to modulate electrophysiological response.

### Evaluative Conditioning and Methodological Limitations

The absence of significant evaluative conditioning across both experiments, with the exception of the aversive imagery condition in Experiment 1, is a surprising result. It is not immediately apparent as to why changes to pleasantness ratings were not observed, and these are contrary to a number of previous evaluative conditioning studies: Petrovic et al. (2008) reported neutral faces paired with electric shock were rated as less sympathetic post-conditioning, similarly Hermans et al. (2002) reported decreased valence ratings for faces paired with electric shock, and Todrank et al. (1995) found decreased valence ratings post-conditioning for faces paired with unpleasant odors. However, both the conditions upon which evaluative conditioning depends, together with a clear understanding of the processes involved have not been entirely resolved in the literature (De Houwer et al., 2001, 2005). Contingency awareness is an important factor that has been identified, with evaluative conditioning found to be more likely to occur when participants are consciously aware of the relationship between the CS and the UCS (De Houwer et al., 2001). For this reason, it may be possible that the current findings may be attributable to insufficient contingency awareness and/or an UCS that was not aversive enough to elicit changes in evaluative conditioning.

In this regard, a limitation of the current study was that no assessment of contingency awareness was included. In light of the debate in the literature regarding whether awareness of CS–UCS association is a necessary condition for evaluative conditioning to occur, in future studies this information would aid interpretation of the current finding regarding unchanged pleasantness ratings post-conditioning. In addition to verbal assessment of evaluative conditioning, implicit measures of evaluative conditioning, such as affective priming (De Houwer, 2007), could additionally be employed in order to provide a more sensitive measure of change.

In regards to investigation of depressive symptomology in association with N170 changes there is also a limitation which could be addressed in future studies. In Experiment 1, the inclusion of participants with depressive symptoms in the mild and moderate range may have been a confounding factor (even though the mean for the sample as a whole was in the minimal range). Also for this reason, the findings from Experiment 1 cannot be interpreted as strictly representing a healthy (non-clinical) adult sample, due to the presence of some evidence of depressive symptoms. To address this limitation in future studies, a more stringent BDI-II exclusion criteria (e.g., BDI scores <10) would be preferable, together with a structured interview to rule out a diagnosis of depression according to DSM criteria, as done in Experiment 2.

## Summary And Future Directions

Taken together, the two experiments provide evidence to suggest that differential classical conditioning using emotional imagery or acoustic startle are capable of enhancing early sensory facial processing, as revealed by amplitude increase in the N170. However, contrary to expectation no differences in the amplitude of the N170 response were observed for individuals with MDD compared to controls. In future research, further investigation regarding the use of positive UCS is also warranted. A direct comparison of N170 amplitudes in response to fearful, sad, happy, and neutral faces, together with measurement of changes to N170 amplitudes for neutral faces following conditioning, would also be of value in answering the question as to whether conditioning results in the same neural responses as those obtained using inherently emotional stimuli. Also in follow-up studies, a more expanded analysis of ERP components including the P100 and the N250 may further elucidate the network of conditioned changes to cortical activity. The use of neuroimaging technique such as fMRI would additionally help to provide complementary spatial information regarding subcortical structures involved in the re-entrant pathways between sensory and association regions. In regards to further investigation of changes to facial processing with differential conditioning in MDD, a more detailed investigation of the relationship between N170 amplitude change and the expression of somatic, cognitive, or affective symptoms would be highly informative.

## Author Contributions

DC was involved in all aspects of the study, both experimental design, analysis, and manuscript preparation; EK was involved in data collection and analysis for Experiment 1; JM was involved in data collection and analysis for Experiment 2; RC was involved in the design and analysis as well as editing of the final manuscript.

## Conflict of Interest Statement

The authors declare that the research was conducted in the absence of any commercial or financial relationships that could be construed as a potential conflict of interest.
