# Supplementary material for: Modulation of the N170 with Classical Conditioning: The Use of Emotional Imagery and Acoustic Startle in Healthy and Depressed Participants
Source: Front Hum Neurosci. 2016 Jun 30;10:337. doi: 10.3389/fnhum.2016.00337 (PMC4928609; doi:10.3389/fnhum.2016.00337)
Supplement: Supplementary file 1 [file Table_1.DOCX]

**SUPPLEMENTARY MATERIALS:**

Table 1: *Experiment 1, nor*mative *valence and arousal mean (SD) data for IAPS images used in the conditioning paradigm.*

|  |  | Valence | | | Arousal | | |
| --- | --- | --- | --- | --- | --- | --- | --- |
| Condition | Gender | M | SD† | range† | M | SD† | range† |
| NEU | *Female* | 5.0 | 0.53 | 4.01 – 5.99 | 4.0 | 0.73 | 2.53 – 5.44 |
|  | *Male* | 5.0 | 0.52 | 4.02 – 5.96 | 4.0 | 0.74 | 2.51 – 5.41 |
| LVHA | *Female* | 2.5 | 0.68 | 1.15 – 4.53 | 6.0 | 0.75 | 3.98 – 7.3 |
|  | *Male* | 2.5 | 0.52 | 1.5 – 3.97 | 6.0 | 0.68 | 4.52 – 7.37 |
| HVHA | *Female* | 7.0 | 0.55 | 6.14 – 7.95 | 6.0 | 0.51 | 5.11 – 6.98 |
|  | *Male* | 7.0 | 0.42 | 6.13 – 7.7 | 6.0 | 0.56 | 5.04 – 6.89 |

*LVHA; Low-valance and high arousal IAPS images, HVHA; high-valence and high arousal IAPS images, NEU: Neutral valence and low arousal IAPS images. †SD and ranges have been calculated across all lists within each condition, whereas the means are maintained for each individual list.*
