# Supplementary material for: Modulation of the N170 with Classical Conditioning: The Use of Emotional Imagery and Acoustic Startle in Healthy and Depressed Participants
Source: Front Hum Neurosci. 2016 Jun 30;10:337. doi: 10.3389/fnhum.2016.00337 (PMC4928609; doi:10.3389/fnhum.2016.00337)
Supplement: Supplementary file 2 [file Table_2.DOCX]

**SUPPLEMENTARY MATERIALS:**

Table 2: *Experiment 1 conditioning paradigm, task conditions according to a 50% reinforcement schedule.*

| Condition | CS+ | UCS | Trials /block |
| --- | --- | --- | --- |
| CS+ /LVHA | Neutral Face 1 | Paired: Aversive IAPS image | 20 |
|  |  | Unpaired: Neutral IAPS image | 20 |
| CS+ /LVHA+ startle | Neutral Face 2 | Paired: Aversive IAPS + startle | 20 |
|  |  | Unpaired: Neutral IAPS image | 20 |
| CS+ /Startle (only) | Neutral Face 3 | Paired: Neutral IAPS + startle | 20 |
|  |  | Unpaired: Neutral IAPS image | 20 |
| CS+ /HVHA | Neutral Face 4 | Paired: Positive IAPS image | 20 |
|  |  | Paired: Neutral IAPS image | 20 |
| CS- /Control | Neutral Face 5 | Neutral IAPS image | 40 |

LVHA; Low-valance high arousal imagery condition, LVHA + startle; Low-valence high arousal imagery and acoustic startle condition, Startle(only); Neutral imagery and acoustic startle condition, HVHA; High-valence high arousal imagery condition, Control; Neutral imagery condition.
