# Supplementary material for: Modulation of the N170 with Classical Conditioning: The Use of Emotional Imagery and Acoustic Startle in Healthy and Depressed Participants
Source: Front Hum Neurosci. 2016 Jun 30;10:337. doi: 10.3389/fnhum.2016.00337 (PMC4928609; doi:10.3389/fnhum.2016.00337)
Supplement: Supplementary file 3 [file Table_3.DOCX]

**SUPPLEMENTARY MATERIALS:**

Table 3: *Experiment 1 face rating subjective pleasantness ratings, before and after conditioning paradigm, by gender.*

|  | | Pleasantness Rating | | | | | |
| --- | --- | --- | --- | --- | --- | --- | --- |
|  | | Before | | After | | Difference | |
|  |  | M | SD | M | SD | M | SD |
| *Male (N=11)* | |  |  |  |  |  |  |
|  | LVHA | 36.636 | (10.376) | 28.545 | (10.511) | -8.091 | (15.997) |
|  | LVHA + Startle | 44.818 | (12.172) | 40.545 | (15.501) | -4.273 | (16.457) |
|  | Startle | 52.364 | (9.646) | 52.000 | (9.808) | -.364 | (10.142) |
|  | HVHA | 49.000 | (12.946) | 47.909 | (13.860) | -1.091 | (10.251) |
|  | Control | 50.091 | (13.420) | 46.727 | (16.249) | -3.364 | (14.320) |
| *Female (N=12)* | |  |  |  |  |  |  |
|  | LVHA | 37.333 | (10.281) | 29.667 | (12.659) | -7.667 | (12.492) |
|  | LVHA + Startle | 41.000 | (13.177) | 45.917 | (15.329) | 4.917 | (15.675) |
|  | Startle | 47.667 | (7.291) | 50.917 | (9.307) | 3.250 | (12.997) |
|  | HVHA | 50.583 | (10.596) | 51.250 | (6.982) | .667 | (9.557) |
|  | Control | 47.917 | (13.474) | 53.333 | (14.028) | 5.417 | (11.317) |
| *All participants (N=23)* | |  |  |  |  |  |  |
|  | LVHA | 37.000 | (10.095) | 29.130 | (11.431) | -7.870 | (13.942) |
|  | LVHA + Startle | 42.826 | (12.569) | 43.348 | (15.305) | .522 | (16.370) |
|  | Startle | 49.913 | (8.639) | 51.435 | (9.346) | 1.522 | (11.603) |
|  | HVHA | 49.826 | (11.531) | 49.652 | (10.705) | -.174 | (9.708) |
|  | Control | 48.957 | (13.186) | 50.174 | (15.159) | 1.217 | (13.318) |
