# Supplementary material for: Modulation of the N170 with Classical Conditioning: The Use of Emotional Imagery and Acoustic Startle in Healthy and Depressed Participants
Source: Front Hum Neurosci. 2016 Jun 30;10:337. doi: 10.3389/fnhum.2016.00337 (PMC4928609; doi:10.3389/fnhum.2016.00337)
Supplement: Supplementary file 4 [file Table_4.DOCX]

**SUPPLEMENTARY MATERIALS:**

Table 4: *Experiment 1 N170 mean amplitude and standard deviations according to time, gender and laterality*

|  |  |  | N170 Amplitude | | | | | | | | | |
| --- | --- | --- | --- | --- | --- | --- | --- | --- | --- | --- | --- | --- |
|  |  |  | LVHA | | LVHA + Startle | | Startle (only) | | HVHA | | Control | |
| *Males (N = 12)* | | | M | SD | M | SD | M | SD | M | SD | M | SD |
|  | P7 | time 1 | -3.204 | (4.082) | -2.462 | (3.994) | -2.784 | (3.764) | -2.978 | (3.963) | -2.117 | (3.513) |
|  |  | time 2 | -3.923 | (3.520) | -2.675 | (3.518) | -3.134 | (4.333) | -2.988 | (3.689) | -2.323 | (4.133) |
|  |  | Total | -3.430 | (3.774) | -2.451 | (3.737) | -2.931 | (3.908) | -2.883 | (3.773) | -2.163 | (3.858) |
|  | P8 | time 1 | -2.682 | (5.931) | -1.718 | (6.132) | -1.769 | (5.633) | -2.082 | (4.582) | -1.661 | (4.811) |
|  |  | time 2 | -3.064 | (4.801) | -1.644 | (5.474) | -2.209 | (5.894) | -2.441 | (5.049) | -1.540 | (5.293) |
|  |  | Total | -2.783 | (5.348) | -1.479 | (5.809) | -1.948 | (5.722) | -2.091 | (4.862) | -1.487 | (5.018) |
| *Females (N=12)* | | |  |  |  |  |  |  |  |  |  |  |
|  | P7 | time 1 | -1.598 | (2.310) | -.744 | (2.297) | -1.134 | (2.328) | -1.395 | (2.361) | -.875 | (1.880) |
|  |  | time 2 | -1.735 | (2.441) | -.954 | (2.473) | -1.586 | (2.290) | -.338 | (2.292) | -.630 | (2.417) |
|  |  | Total | -1.589 | (2.176) | -.811 | (2.316) | -1.290 | (2.239) | -.769 | (2.221) | -.671 | (1.986) |
|  | P8 | time 1 | -1.667 | (5.784) | -1.863 | (4.887) | -2.119 | (5.550) | -2.544 | (5.529) | -1.493 | (5.151) |
|  |  | time 2 | -1.964 | (5.071) | -1.847 | (4.656) | -2.489 | (5.617) | -1.823 | (5.521) | -1.469 | (4.982) |
|  |  | Total | -1.770 | (5.382) | -1.810 | (4.669) | -2.245 | (5.568) | -2.145 | (5.519) | -1.435 | (5.049) |
| *All participants (N=24)* | | |  |  |  |  |  |  |  |  |  |  |
|  | P7 | time 1 | -2.401 | (3.345) | -1.603 | (3.305) | -1.959 | (3.175) | -2.187 | (3.291) | -1.496 | (2.827) |
|  |  | time 2 | -2.829 | (3.166) | -1.814 | (3.101) | -2.360 | (3.480) | -1.663 | (3.294) | -1.477 | (3.422) |
|  |  | Total | -2.510 | (3.156) | -1.631 | (3.153) | -2.111 | (3.225) | -1.826 | (3.214) | -1.417 | (3.096) |
|  | P8 | time 1 | -2.175 | (5.753) | -1.790 | (5.423) | -1.944 | (5.472) | -2.313 | (4.972) | -1.577 | (4.875) |
|  |  | time 2 | -2.514 | (4.862) | -1.746 | (4.971) | -2.349 | (5.632) | -2.132 | (5.183) | -1.505 | (5.027) |
|  |  | Total | -2.277 | (5.273) | -1.645 | (5.157) | -2.096 | (5.523) | -2.118 | (5.087) | -1.461 | (4.923) |
