# Supplementary material for: Modulation of the N170 with Classical Conditioning: The Use of Emotional Imagery and Acoustic Startle in Healthy and Depressed Participants
Source: Front Hum Neurosci. 2016 Jun 30;10:337. doi: 10.3389/fnhum.2016.00337 (PMC4928609; doi:10.3389/fnhum.2016.00337)
Supplement: Supplementary file 5 [file Table_5.DOCX]

**SUPPLEMENTARY MATERIALS:**

Table 5: *Experiment 2 normative valence and arousal mean (SD) data for the IAPS images used in the conditioning paradigm.*

|  |  |  | Valence | | | Arousal | | |
| --- | --- | --- | --- | --- | --- | --- | --- | --- |
| Condition | Gender | N^†^ | M | SD | range | M | SD | range |
| Neutral | *Male* | 40 | 5.00 | (0.26) | 0.93 | 3.01 | (0.46) | 1.69 |
|  | *Female* | 40 | 5.00 | (0.22) | 0.89 | 3.04 | (0.42) | 1.60 |
|  | *Total* | 80 | 5.00 | (0.24) | 0.93 | 3.02 | (0.44) | 1.70 |
| Negative | *Male* | 40 | 2.99 | (0.53) | 1.88 | 5.98 | (0.53) | 1.89 |
|  | *Female* | 40 | 3.01 | (0.59) | 1.97 | 5.99 | (0.56) | 1.85 |
|  | *Total* | 80 | 3.00 | (0.55) | 1.97 | 5.98 | (0.54) | 1.96 |
| Positive | *Male* | 40 | 6.97 | (0.42) | 1.70 | 5.97 | (0.59) | 1.90 |
|  | *Female* | 40 | 6.98 | (0.58) | 1.82 | 6.01 | (0.51) | 1.93 |
|  | *Total* | 80 | 6.97 | (0.50) | 1.82 | 5.99 | (0.55) | 1.93 |

^†^ *Number of images across the two presentation blocks*
