# Supplementary material for: Modulation of the N170 with Classical Conditioning: The Use of Emotional Imagery and Acoustic Startle in Healthy and Depressed Participants
Source: Front Hum Neurosci. 2016 Jun 30;10:337. doi: 10.3389/fnhum.2016.00337 (PMC4928609; doi:10.3389/fnhum.2016.00337)
Supplement: Supplementary file 6 [file Table_6.DOCX]

**SUPPLEMENTARY MATERIALS:**

Table 6: *Experiment 2 imaging Rating means (SD) by gender and group (control, depressed; N=30)*

|  | |  |  | Valence | | Arousal | |
| --- | --- | --- | --- | --- | --- | --- | --- |
|  | Valence | Gender | N | M | SD | M | SD |
| Control | | | | | | | |
|  | *Neutral* | Male | 7 | 5.14 | (0.25) | 3.09 | (1.35) |
|  | | Female | 12 | 5.04 | (0.16) | 3.66 | (1.10) |
|  | | Combined | 19 | 5.08 | (0.20) | 3.45 | (1.20) |
|  | *Negative* | Male | 7 | 2.97 | (0.83) | 5.87 | (1.44) |
|  | | Female | 12 | 3.28 | (0.80) | 5.65 | (1.04) |
|  | | Combined | 19 | 3.17 | (0.81) | 5.73 | (1.17) |
|  | *Positive* | Male | 7 | 6.35 | (0.66) | 5.73 | (1.56) |
|  | | Female | 12 | 6.42 | (0.67) | 5.70 | (0.90) |
|  | | Combined | 19 | 6.39 | (0.65) | 5.71 | (1.15) |
| Depressed | | | | | | | |
|  | *Neutral* | Male | 4 | 5.08 | (0.27) | 2.21 | (1.05) |
|  | | Female | 7 | 4.99 | (0.32) | 3.33 | (1.37) |
|  | | Combined | 11 | 5.03 | (0.29) | 2.92 | (1.33) |
|  | *Negative* | Male | 4 | 3.40 | (1.01) | 3.31 | (1.67) |
|  | | Female | 7 | 3.24 | (0.69) | 5.44 | (1.31) |
|  | | Combined | 11 | 3.30 | (0.77) | 4.66 | (1.74) |
|  | *Positive* | Male | 4 | 5.68 | (1.18) | 3.36 | (0.88) |
|  | | Female | 7 | 5.80 | (0.63) | 4.74 | (1.51) |
|  | | Combined | 11 | 5.76 | (0.81) | 4.24 | (1.44) |
