# Supplementary material for: Modulation of the N170 with Classical Conditioning: The Use of Emotional Imagery and Acoustic Startle in Healthy and Depressed Participants
Source: Front Hum Neurosci. 2016 Jun 30;10:337. doi: 10.3389/fnhum.2016.00337 (PMC4928609; doi:10.3389/fnhum.2016.00337)
Supplement: Supplementary file 7 [file Table_7.DOCX]

**SUPPLEMENTARY MATERIALS:**

Table 7: *Experiment 2 face rating subjective pleasantness ratings, before and after conditioning paradigm, by gender.*

|  | |  |  | Before | | After | | Difference | |
| --- | --- | --- | --- | --- | --- | --- | --- | --- | --- |
|  | Valence | Gender | N | M | SD | M | SD | M | SD |
| Control | | | | | | | |  |  |
|  | *Neutral* | Male |  | 55.50 | (15.88) | 53.00 | (11.28) | -2.50 | (11.29) |
|  | | Female |  | 50.93 | (8.24) | 50.50 | (9.61) | -0.43 | (8.71) |
|  | | Combined |  | 52.59 | (11.45) | 51.41 | (10.05) | -1.18 | (9.51) |
|  | *Negative* | Male |  | 45.25 | (9.29) | 49.38 | (12.41) | 4.13 | (15.70) |
|  | | Female |  | 51.50 | (11.12) | 56.43 | (12.97) | 4.93 | (11.61) |
|  | | Combined |  | 49.23 | (10.71) | 53.86 | (12.94) | 4.64 | (12.87) |
|  | *Positive* | Male |  | 48.88 | (16.82) | 48.75 | (14.61) | -0.13 | (8.51) |
|  | | Female |  | 50.43 | (10.05) | 50.79 | (12.39) | 0.36 | (15.29) |
|  | | Combined |  | 49.86 | (12.55) | 50.05 | (12.93) | 0.18 | (12.99) |
| Depressed | | | | | | | |  |  |
|  | *Neutral* | Male |  | 41.75 | (9.60) | 43.25 | (7.93) | 1.50 | (5.07) |
|  | | Female |  | 44.92 | (14.78) | 46.38 | (15.77) | 1.46 | (8.29) |
|  | | Combined |  | 44.18 | (13.53) | 46.65 | (14.15) | 1.47 | (7.51) |
|  | *Negative* | Male |  | 42.50 | (9.95) | 47.75 | (4.79) | 5.25 | (12.04) |
|  | | Female |  | 41.62 | (17.37) | 42.08 | (19.42) | 0.46 | (14.71) |
|  | | Combined |  | 41.82 | (15.65) | 43.41 | (17.12) | 1.59 | (13.92) |
|  | *Positive* | Male |  | 48.00 | (18.60) | 43.50 | (6.86) | -4.50 | (22.58) |
|  | | Female |  | 36.92 | (21.29) | 40.38 | (22.22) | 3.46 | (13.61) |
|  | | Combined |  | 39.53 | (20.69) | 41.12 | (19.52) | 1.59 | (15.70) |
