# Supplementary material for: Modulation of the N170 with Classical Conditioning: The Use of Emotional Imagery and Acoustic Startle in Healthy and Depressed Participants
Source: Front Hum Neurosci. 2016 Jun 30;10:337. doi: 10.3389/fnhum.2016.00337 (PMC4928609; doi:10.3389/fnhum.2016.00337)
Supplement: Supplementary file 8 [file Table_8.DOCX]

**SUPPLEMENTARY MATERIALS:**

Table 8: *Experiment 2, N170 mean (SD) amplitudes by gender, condition, laterality.*

|  |  |  |  | Neutral | | Negative | | Positive | |
| --- | --- | --- | --- | --- | --- | --- | --- | --- | --- |
|  | | | | M | SD | M | SD | M | SD |
| *Controls* | | | |  |  |  |  |  |  |
|  | *Males (N = 9)* | | |  |  |  |  |  |  |
|  |  | P7 | baseline | 0.14 | (2.86) | 0.59 | (3.38) | 0.03 | (3.23) |
|  |  |  | block 1 | -0.54 | (2.24) | -1.07 | (2.75) | -1.26 | (3.42) |
|  |  |  | block 2 | -0.63 | (3.53) | -0.80 | (4.31) | -1.33 | (3.17) |
|  |  | P8 | baseline | 0.86 | (4.19) | 0.70 | (5.14) | 0.52 | (4.70) |
|  |  |  | block 1 | -1.16 | (4.31) | -1.51 | (4.80) | -1.36 | (4.59) |
|  |  |  | block 2 | -1.87 | (5.11) | -1.89 | (5.02) | -1.39 | (4.48) |
|  | *Females (N=17)* | | |  |  |  |  |  |  |
|  |  | P7 | baseline | -0.16 | (2.43) | 0.27 | (3.37) | 0.25 | (2.72) |
|  |  |  | block 1 | -0.54 | (2.22) | -1.04 | (3.06) | -1.28 | (2.72) |
|  |  |  | block 2 | -0.62 | (2.83) | -0.39 | (2.81) | -0.85 | (2.05) |
|  |  | P8 | baseline | 0.98 | (4.17) | 1.29 | (3.94) | 1.37 | (4.38) |
|  |  |  | block 1 | 0.20 | (3.24) | -0.19 | (4.21) | -0.39 | (4.33) |
|  |  |  | block 2 | -0.04 | (3.55) | 0.32 | (3.83) | -0.48 | (3.16) |
|  | *Combined (N=26)* | | |  |  |  |  |  |  |
|  |  | P7 | baseline | -0.06 | (2.54) | 0.38 | (3.31) | 0.17 | (2.84) |
|  |  |  | block 1 | -0.54 | (2.18) | -1.05 | (2.90) | -1.27 | (2.91) |
|  |  |  | block 2 | -0.62 | (3.02) | -0.53 | (3.32) | -1.01 | (2.44) |
|  |  | P8 | baseline | 0.94 | (4.09) | 1.09 | (4.30) | 1.07 | (4.42) |
|  |  |  | block 1 | -0.27 | (3.62) | -0.64 | (4.37) | -0.73 | (4.35) |
|  |  |  | block 2 | -0.68 | (4.14) | -0.44 | (4.31) | -0.79 | (3.60) |
| *Depressed* | | | | | | | | | |
|  | *Males (N = 5)* | | | | | | | | |
|  |  | P7 | baseline | 0.99 | (3.76) | 1.39 | (2.37) | 0.94 | (3.58) |
|  |  |  | block 1 | 0.18 | (2.70) | -0.11 | (2.36) | -0.76 | (2.68) |
|  |  |  | block 2 | 0.18 | (2.59) | 0.43 | (2.90) | -1.25 | (2.64) |
|  |  | P8 | baseline | 0.69 | (5.06) | 0.11 | (3.70) | 0.07 | (4.11) |
|  |  |  | block 1 | -1.52 | (4.93) | -0.85 | (4.37) | -1.22 | (4.76) |
|  |  |  | block 2 | 0.01 | (4.41) | -0.86 | (6.05) | -1.48 | (5.49) |
|  | *Females (N=13)* | | | | | | | | |
|  |  | P7 | baseline | -0.63 | (3.65) | -0.60 | (4.07) | -0.54 | (3.94) |
|  |  |  | block 1 | -0.42 | (3.49) | -1.94 | (3.65) | -1.26 | (3.35) |
|  |  |  | block 2 | -0.64 | (3.72) | -1.28 | (3.29) | -0.65 | (2.90) |
|  |  | P8 | baseline | 0.70 | (4.88) | 0.01 | (5.19) | -0.09 | (5.54) |
|  |  |  | block 1 | -0.31 | (4.20) | -1.17 | (4.39) | -1.35 | (4.51) |
|  |  |  | block 2 | -0.26 | (3.90) | -1.75 | (3.99) | -0.74 | (4.10) |

Table 8 continued: *Experiment 2, N170 mean (SD) amplitudes by gender, condition, laterality, continued…*

|  |  |  |  | Neutral | | Negative | | Positive | |
| --- | --- | --- | --- | --- | --- | --- | --- | --- | --- |
|  | | | | M | SD | M | SD | M | SD |
| *Depressed* | | | |  |  |  |  |  |  |
|  | *Combined (N=18)* | | |  |  |  |  |  |  |
|  |  | P7 | baseline | -0.18 | (3.64) | -0.05 | (3.72) | -0.13 | (3.80) |
|  |  |  | block 1 | -0.25 | (3.23) | -1.43 | (3.38) | -1.12 | (3.11) |
|  |  |  | block 2 | -0.41 | (3.39) | -0.81 | (3.20) | -0.82 | (2.77) |
|  |  | P8 | baseline | 0.70 | (4.78) | 0.04 | (4.71) | -0.05 | (5.06) |
|  |  |  | block 1 | -0.64 | (4.30) | -1.08 | (4.26) | -1.32 | (4.44) |
|  |  |  | block 2 | -0.19 | (3.92) | -1.51 | (4.47) | -0.95 | (4.37) |
| *All participants* | | | | | | | | | |
|  | *Males (N = 14)* | | | | | | | | |
|  |  | P7 | baseline | 0.44 | (3.09) | 0.87 | (2.99) | 0.35 | (3.25) |
|  |  |  | block 1 | -0.29 | (2.34) | -0.72 | (2.57) | -1.08 | (3.08) |
|  |  |  | block 2 | -0.34 | (3.15) | -0.36 | (3.79) | -1.30 | (2.89) |
|  |  | P8 | baseline | 0.80 | (4.32) | 0.49 | (4.53) | 0.36 | (4.34) |
|  |  |  | block 1 | -1.29 | (4.35) | -1.27 | (4.49) | -1.31 | (4.47) |
|  |  |  | block 2 | -1.20 | (4.79) | -1.52 | (5.20) | -1.42 | (4.65) |
|  | *Females (N=30)* | | | | | | | | |
|  |  | P7 | baseline | -0.37 | (2.97) | -0.11 | (3.65) | -0.09 | (3.26) |
|  |  |  | block 1 | -0.49 | (2.79) | -1.43 | (3.30) | -1.27 | (2.95) |
|  |  |  | block 2 | -0.63 | (3.19) | -0.78 | (3.01) | -0.76 | (2.41) |
|  |  | P8 | baseline | 0.86 | (4.41) | 0.73 | (4.49) | 0.74 | (4.88) |
|  |  |  | block 1 | -0.02 | (3.63) | -0.61 | (4.24) | -0.81 | (4.36) |
|  |  |  | block 2 | -0.14 | (3.64) | -0.58 | (3.97) | -0.59 | (3.54) |
|  | *Combined (N=44)* | | | | | | | | |
|  |  | P7 | baseline | -0.11 | (3.00) | 0.20 | (3.45) | 0.05 | (3.23) |
|  |  |  | block 1 | -0.42 | (2.63) | -1.21 | (3.07) | -1.21 | (2.96) |
|  |  |  | block 2 | -0.54 | (3.14) | -0.64 | (3.24) | -0.93 | (2.55) |
|  |  | P8 | baseline | 0.84 | (4.33) | 0.66 | (4.45) | 0.62 | (4.67) |
|  |  |  | block 1 | -0.42 | (3.87) | -0.82 | (4.28) | -0.97 | (4.35) |
|  |  |  | block 2 | -0.48 | (4.01) | -0.88 | (4.36) | -0.86 | (3.89) |
