# Supplementary material for: Modulation of the N170 with Classical Conditioning: The Use of Emotional Imagery and Acoustic Startle in Healthy and Depressed Participants
Source: Front Hum Neurosci. 2016 Jun 30;10:337. doi: 10.3389/fnhum.2016.00337 (PMC4928609; doi:10.3389/fnhum.2016.00337)
Supplement: Supplementary file 9 [file Table_9.DOCX]

**SUPPLEMENTARY MATERIALS:**

Table 9: *Experiment 1, N170 main effects and interactions*

The two main effects for condition that were associated with the two sets of contrasts are as follows:

F(2,92) = 6.847, p=.003, partial eta = 0.157. Condition has 5 levels in this analysis (all CS+ conditions)

F(2,46) = 6.969, p=.024, partial eta = 0.150. Condition has only 3 levels in this analysis (restricted to aversive conditions)
