# Supplementary material for: Modulation of the N170 with Classical Conditioning: The Use of Emotional Imagery and Acoustic Startle in Healthy and Depressed Participants
Source: Front Hum Neurosci. 2016 Jun 30;10:337. doi: 10.3389/fnhum.2016.00337 (PMC4928609; doi:10.3389/fnhum.2016.00337)
Supplement: Supplementary file 10 [file Table_10.DOCX]

**SUPPLEMENTARY MATERIALS:**

Table 10: *Experiment 2, N170 main effects and interactions*

Main effects for block: F(2,84)=20.719,p<.001, partial eta^2^=0.330)

Main effect for condition: F(2,84)= 2.511,p=.087,partial eta^2^=.056)

Main effect for laterality: F(1,42) = .380, p=.541, partial eta^2^=.009)

Main effect for group: F(1,42) = .085, p=.772, partial eta^2^ = .002)

Group x condition interaction: F(2,84) = 1.511, p=.227, partial eta^2^ = .035)

Group x condition x block interaction: F(4,168)= 0.537,p=.708, partial eta^2^ = .013)

laterality x block interaction: F(2,84) = 4.194, p =.018, partial eta^2^ = .091)

condition x block interaction: F(4,168) = 1.555, p=.189,partial eta^2^=.036)

condition x block x laterality interaction: F(4,168) = 2.608, p=.037, partial eta^2^=.058)

condition x block x laterality x group interaction: F(4,168)= 1.593,p=.178, partial eta^2^=.037)
